# Utility of Novel Hypochromia and Microcythemia Markers in Classifying Hematological and Iron Status in Male Athletes

**DOI:** 10.3390/nu11112767

**Published:** 2019-11-14

**Authors:** Jadwiga Malczewska-Lenczowska, Olga Surała, Joanna Orysiak, Dariusz Turowski, Beata Szczepańska, Paweł Tomaszewski

**Affiliations:** 1 Department of Nutrition Physiology and Dietetics, Institute of Sport, National Research Institute, Trylogii 2/16, 01-982 Warsaw, Poland; olga.surala@insp.waw.pl (O.S.); joanna.orysiak@insp.waw.pl (J.O.); beata.szczepanska@insp.waw.pl (B.S.); 2 Department of Biochemistry, Institute of Sport, National Research Institute, Trylogii 2/16, 01-982 Warsaw, Poland; dariusz.turowski@insp.waw.pl; 3 Department of Biometry, Józef Piłsudski University of Physical Education, Marymoncka 34, 00-968 Warsaw, Poland

**Keywords:** iron deficiency, sports anemia, hypochromia and microcythemia indices, reticulocyte, erythrocyte, male athletes

## Abstract

In athletes, no reliable indices exist for an unambiguous evaluation of hematological and iron status. Therefore, the utility of some new red blood cell (RBC) parameters was explored in 931 elite male athletes aged 13–35 years. To diagnose iron status, the values of ferritin and soluble transferrin receptor (sTfR), total iron binding capacity (TIBC), and basic blood morphology were determined in blood. The new hematological markers included among others: mean cellular hemoglobin content in reticulocytes (CHr), percentage of erythrocytes (HYPOm) and reticulocytes (HYPOr) with decreased cellular hemoglobin concentration, percentage of erythrocytes (LowCHm) and reticulocytes (LowCHr) with decreased cellular hemoglobin content, mean volume of reticulocytes (MCVr), and percentage of erythrocytes with decreased volume (MICROm). Despite adverse changes in reticulocyte hypochromia indices (CHr, LowCHr, HYPOr; *p* < 0.001) in the iron depletion state, the area under the receiver operating characteristic curve (AUC-ROC) values calculated for them were relatively low (0.539–0.722). In iron-deficient erythropoiesis (IDE), unfavorable changes additionally concern microcythemia indices in both reticulocytes and erythrocytes (MCVr, MCV, MICROm, and red cell volume distribution width—RDW), with especially high values of AUC-ROC (0.947–0.970) for LowCHm, LowCHr, and CHr. Dilutional sports anemia was observed in 6.1% of athletes. In this subgroup, only hemoglobin concentration (Hb), hematocrit (Hct), and RBC (all dependent on blood volume) were significantly lower than in the normal group. In conclusion, the diagnostic utility of the new hematology indices was not satisfactory for the detection of an iron depletion state in athletes. However, these new indices present high accuracy in the detection of IDE and sports anemia conditions.

## 1. Introduction

Iron is a dominant mineral in processes involving oxygen transport and energy metabolism. Therefore, among athletes, maintaining a normal iron status is a crucial factor contributing to physical capacity [1]. Iron status reflects the balance between the rate of erythropoiesis and the size of the body iron stores [2]. In athletic populations, the reserve pool of body iron may diminish due to many factors: increased iron losses via hemolysis, sweating, gastrointestinal bleeding, urinary blood losses [3], post-exercise increase of hepcidin concentration [4], insufficient dietary iron intake [5], and menstrual blood loss in females [6]. For these reasons, athletes are at a higher risk of experiencing iron deficiency (ID) than non-athletic populations.

The assessment of iron status presents some methodological challenges in sport. For example, physical exertion can promote an inflammatory state, which, in turn, may affect some biochemical indicators of iron metabolism, such as ferritin, transferrin, and iron [1,7,8], so in athletes, the results may be falsely changed. Furthermore, physical exercise may induce changes in plasma volume (in both directions) that impact the interpretation of hematology indices, especially those indicators calculated per unit of blood volume [9,10]. Despite the availability of many blood indices of iron status, no reliable markers exist for the unambiguous detection of ID.

In recent years, new indices of red blood cells have emerged in parallel with technological developments, including both erythrocytes and maturing cells (i.e., reticulocytes). These new parameters, measured simultaneously with basic blood morphology, are helpful in assessing iron status in healthy subjects [10,11,12,13,14,15] and patients with various disorders [2,16,17]. Due to the short life span of reticulocytes (i.e., 24–48 h), markers of maturing red blood cells offer some diagnostic value in assessing subclinical ID [2,18,19]. The most frequently studied reticulocyte marker is cellular hemoglobin content (CHr), as it provides an estimate of recent functional availability of iron to erythron, before the mature cell indices move below the reference intervals [14,20]. Moreover, CHr is free from biological variability and is not influenced by inflammation and infection [11]. For these reasons, many studies have investigated the diagnostic utility of CHr among subjects with different acute or chronic diseases [11,16,21,22,23,24]. Since rigorous physical exercise may also stimulate inflammation [1,4], this parameter could add value in diagnosing iron status in athletic populations. To date, few studies have examined the utility of CHr as an assessment tool in sport [25,26,27]. The most commonly accepted CHr cut-off value for anemia is 28 pg [12,13,20], but no standardized threshold exists that would allow a robust diagnosis of this condition. Adding to this, much less is known about the CHr cut-off values for detecting the two stages of latent iron deficiencies, i.e., iron depletion and iron-deficient erythropoiesis. The limited reports in this area have examined both stages together [14,19], and there is a clear lack of studies, especially on athletes who, we argue, are likely to present a broad spectrum of iron-deficient states across different sports and levels of participation [28,29,30]. Although athletes are generally considered to be healthy subjects, some suggest that it is inappropriate to use normal clinical reference values when interpreting an athlete’s biochemical and hematological status [27,31]. Thus, targeted research on athletes who are exposed to acute and chronic physical exercise is needed.

Because reticulocyte parameters exhibit only minor changes under physical training [26], other indices of maturing red blood cells could have diagnostic utility in sport. Our recent study demonstrated visible changes in many reticulocyte indices among female athletes already at a very early stage of ID [25]. Furthermore, other novel indicators of mature red blood cells are emerging as reliable tests for detecting ID before overt anemia is present, in both athletic [25,26] and non-athletic cohorts [19,23], although their reference intervals have not been established. Subsequently, the inclusion of new erythrocyte indices, especially those independent from blood volume, could also prove fruitful in the assessment of hematological status in athletes.

Given the fundamental role of iron for health and physical performance, increased risk of its deficiency in athletes, and due to difficulties in the correct evaluation of iron status based on conventional indices, it is necessary to look for new hematological indicators, especially since, so far, their use in assessing hematological and iron status in this group is relatively limited. Furthermore, to our knowledge, there are no studies assessing the prevalence of dilutional sports anemia among athletes. Meanwhile, the phenomenon often makes the proper evaluation of the hematological status in physically active subjects difficult. For this reason, we aimed to analyze the diagnostic utility of novel hypochromia and microcythemia markers in the detection of ID and assessment of its progression as well as in classifying hematological status in male athletes.

## 2. Material and Methods

### 2.1. Subjects

This research is retrospective. Initially, blood test results of 994 male professional athletes at the national level were analyzed. The blood samples were withdrawn on the occasion of the periodic medical examination in the years 2014–2016. The athletes were in various phases of the training cycle. In order to rule out factors that may have had a potential impact on indices of iron status, 2 exclusion criteria were applied. The first criterion was the presence of any symptoms of an acute-phase reaction, expressed by increased values of erythrocyte sedimentation rate (ESR) or C-reactive protein concentration (CRP) or white blood cell count (WBC). The symptoms of post-training fatigue expressed in elevated activities of creatine kinase, and 3 liver enzymes, i.e., alanine aminotransferase (ALT), aspartate aminotransferase (AST), and gamma glutamyl transpeptidase (GGT) were used as a second exclusion criterion. Using these two criteria allowed for the minimization of false results with respect to diagnosing iron deficiency. Finally, only the single results of 931 healthy male athletes (aged 13.0–35.1 years) were taken for statistical analysis. The athletes represented the following sport disciplines: cycling (*n* = 141), rowing (*n* = 111), canoeing (*n* = 91), ice hockey (*n* = 86), swimming (*n* = 81), volleyball (*n* = 62), middle and long distance running (*n* = 42), wrestling (*n* = 38), handball (*n* = 36), cross-country skiing, (*n* = 31), boxing (*n* = 27), Nordic combined skiing (*n* = 21), judo (*n* = 20), tennis (*n* = 27), modern pentathlon (*n* = 26), sailing (*n* = 25), field hockey (*n* = 16), speed skating (*n* = 19), water polo (*n* = 13), fencing (*n* = 10), and triathlon (*n* = 8). The data regarding iron intake in the diet or iron supplementation were not collected from the participants. The study was performed according to the Declaration of Helsinki and approved by the local ethics committee (protocols: KEBN-19-47-JM). All subjects gave their informed consent for inclusion before testing. Written informed consent was obtained from participants or their parents if the subject was under 18 years of age. Basic data concerning characteristics of studied athletes are presented in Table 1.

### 2.2. Blood Analysis

To assess iron status, the blood was withdrawn from the antecubital vein in the morning (between 8 and 9 a.m.) in the pre-prandial state, after overnight fasting, and a minimum of 12 h after the last training session. To eliminate any residual effect of physical movement and ensure the data collected reflected a resting baseline, a sample collection started after about 10 min rest in a seated position. The following measurements were performed in whole blood regarding mature erythrocytes: hemoglobin concentration (Hb), hematocrit (Hct), red blood cell count (RBC), mean corpuscular hemoglobin concentration (MCHC), mean corpuscular volume (MCV), mean corpuscular hemoglobin (MCH), mean cellular hemoglobin content (CH), percentage of erythrocytes with decreased cellular hemoglobin concentration (%HYPOm), percentage of erythrocytes with decreased cellular hemoglobin content (%LowCHm), percentage of erythrocytes with decreased volume (%MICROm), and red cell volume distribution width (RDW), as well as regarding reticulocytes: mean cellular hemoglobin content (CHr), reticulocyte count expressed as an absolute number (#RET) and as a percentage of the absolute value (%RET), cellular hemoglobin concentration mean (CHCMr), mean corpuscular volume (MCVr), percentage of reticulocytes with decreased cellular hemoglobin concentration (%HYPOr), and percentage of reticulocyte population with decreased cellular hemoglobin content (%LowCHr). The hematological analysis was performed up to 3 h after blood collection using the ADVIA 120 hematology system (Siemens Healthcare, Erlangen, Germany). In our laboratory within-run precision of the hematological parameters, expressed as coefficient of variations (CV), obtained from 20 repetitions of the same blood sample was as follows: Hb 1.26%; Hct 1.01%; RBC 0.93%; MCHC 0.88%; MCV 0.15%; MCH 0.89%; CH 0.19; RDW 0.98%; %HYPOm 13.8%; %LowCHm 1.72%; %MICROm 6.68%; CHr 0.31%; #RET 5.05%; %RET 5.31%; CHCMr 0.62%; MCVr 0.69%; %HYPOr 21.4%, and %LowCHr 21.4%.

In serum, the following analyses were conducted: soluble transferrin receptor (sTfR) concentration using immunoenzymatic commercial kits (Ramco, Stafford, TX, USA); ferritin concentration using an immunoturbidimetric method, iron concentration and total iron binding capacity (TIBC) using a colorimetric method (Pentra, Horiba, ABX, Montpellier Cedex 4-France). Inter-assay variability for those indices did not exceed 6.0%, 7.5%, 4.3%, and 3.9% respectively.

To assess the acute phase reaction, WBC (ADVIA 120 hematology system, Siemens Healthcare, Erlangen, Germany), ESR after one hour in whole blood, and CRP in serum (immunoturbidimetric method, Pentra, Horiba ABX, Montpellier Cedex 4-France) were determined. The CRP reagent used in the study covered a wide range of linearity containing both normal (1.0–5.0 mg/L) and inflammatory response ranges (5.0–160.0 mg/L). Reproducibility of the test performance (between-run precision) did not exceed 4.3%. All serum assays were performed in never-frozen or only once-frozen (−20 ℃) serum samples. All analyses were done in a laboratory with an implemented quality system at the Institute of Sport, National Research Institute (protocol of accreditation #AB946).

Body iron stores were calculated based on the results of ferritin and sTfR concentrations using a special algorithm, which was developed by Cook [32] exclusively for sTfR determined by the Ramco method.

The definition of ID was based on ferritin, sTfR, TIBC, and 3 basic morphological indices, i.e., Hb, RBC and Hct. To classify the respective stages of ID in the male athletic population, the following criteria were applied:

Stage 1—iron depletion: iron stores in bone marrow, liver, and spleen are decreased or exhausted (ferritin concentration < 30 µg/L, TIBC < 410 µg/dL, sTfR < 8.3 mg/L, Hb, Hct, and RBC within the reference range;

Stage 2—iron-deficient erythropoiesis (IDE): iron supply to the erythron is reduced (ferritin concentration < 30 µg/L, TIBC > 410 µg/dL, and/or sTfR > 8.3 mg/L (with the normal reticulocyte count), Hb, Hct, and RBC within the reference range;

Stage 3—iron deficiency anemia (IDA): the amount of hemoglobin falls, resulting in anemia (ferritin concentration < 15 µg/L, TIBC > 410 µg/dL, sTfR > 8.3 mg/L, Hb concentration (<13 g/L) and/or Hct value (<42%) and/or RBC count (<4.7 × 10^12^/L).

Subjects with the value of ferritin lower than 30 µg/L were taken for classification of both subclinical iron deficiencies [33,34], while the levels below 15 µg/L are considered diagnostic for IDA [25,26,27,28,29,30,31,32,33,34,35,36,37]. Due to low specificity and a wide diurnal variation of iron concentration, this indicator and transferrin saturation were not taken into account as criteria of iron deficiency [2].

Sports anemia was recognized in athletes without any symptoms of iron deficiency in whom at least one of 3 basic hematological indices (Hb, Hct, and RBC) were below reference values.

### 2.3. Statistical Analysis

Basic statistical measures were used to describe the results: arithmetic means, standard deviations, ranges, and percentages. Normality of the distributions and the equality of variances were verified using the Shapiro-Wilk test and Levene’s test, respectively. Due to severe violations of assumptions of the parametric procedures, nonparametric tests were used in the data analysis. The differences between normal iron status and iron deficiency (ID and IDE) subgroups were tested using the Kruskal-Wallis rank test, followed by Dunn’s procedure. The Mann-Whitney U test was used to assess the differences between subgroups of sports anemia and athletes with normal iron status. In all analyses, the d-equivalent measure was calculated to express the effect size [38]. Interpretation of effect size was based on Cohen’s classification where d = 0.2 is “small”, d = 0.5 “medium,” and d = 0.8 reflects a “large” effect [39]. Receiver operating characteristic (ROC) curve analysis was used to evaluate the diagnostic performance of selected hematological and iron metabolism indices in the assessment of two latent stages of iron deficiency, and ROC-AUC (area under ROC curves) were drawn for them. Cut-off values were established according to the optimal ratio of sensitivity and specificity. Calculations were performed using the STATISTICA v. 13.0 statistical software package (TIBCO, Palo Alto, CA, USA). In all analyses, the level of significance was set at 0.05.

## 3. Results

Iron deficiency was observed in 23.1% of studied male athletes, wherein the majority of ID cases were latent. Iron depletion was observed in 20.5%, and the second stage (iron-deficient erythropoiesis) in 2.3% of male athletes. Iron deficiency anemia was detected in three subjects only, which represented 0.3% of the studied group.

The iron status and hematological indices in the four subgroups of male athletes, i.e., with normal iron status, sports anemia, iron depletion, and iron-deficient erythropoiesis (IDE), are presented in Table 2.

Because ferritin concentration was used as a basic parameter in classifying iron deficiency, the mean values of this marker were automatically significantly lower (*p* < 0.001) in both groups with iron deficiency. In comparison to athletes with normal iron status, the subjects with stage I ID differed in almost all analyzed indicators (with the exception of the number of reticulocytes and erythrocytes as well as MCV). However, large effects (d-equivalent > 0.8) were observed for only two indicators, i.e., ferritin (64.1 ± 32.0 µg/L vs. 22.6 ± 4.9 µg/L) and body iron (7.55 ± 1.86 mg/kg vs. 3.57 ± 1.44 mg/kg). Among other biochemical, indices both subgroups differed (with medium effect) in TIBC, although the values of this parameter remained within the reference range. With regards to hematological indices, significant differences were mostly observed for reticulocyte parameters, i.e., CHCMr, CHr, %LowCHr, %HYPOr, while among mature red blood cells both subgroups were varied in terms of %LowCHm, CH, and RDW. In the iron depletion subgroup, the values of CHCMr, CHr, and CH were significantly lower (*p* < 0.001), while percentages of HYPOr, LowCHr, LowCHm, and RDW were significantly higher (*p* < 0.001), showing a medium effect size (0.55–0.65) in comparison to athletes with normal iron status.

In subjects with IDE, the differences in biochemical iron-related indices in relation to the normal iron status subgroup were more profound and concerned not only lower ferritin concentration (*p* < 0.001) and higher TIBC value (*p* < 0.001) but also higher sTfR concentration (*p* < 0.001). The athletes with iron-deficient erythropoiesis had a significantly lower (*p* < 0.001) relative value of body iron (−0.67 ± 3.11 mg/kg). With regards to hematological indices, significant differences (*p* < 0.001) with a simultaneously medium effect size (0.50–0.60) between these two subgroups were observed in the following indicators of reticulocytes: CHr, CHCMr, %LowCHr, and %HYPOr, and of erythrocytes: MCH, MCV, CH, %MICROm, and %LowCHm. In athletes with stage II ID, the values of CHr, CHCMr, MCH, MCV, and CH were lower, while percentages of LowCHr, HYPOr, LowCHm, and MICROm were higher (Table 2).

Differences between the two subgroups with iron deficiency, i.e., iron depletion and IDE, were observed for both hematological and biochemical indices. Highly significant differences (*p* < 0.001) with a medium effect size (from 0.51 to 0.73) were observed for the following parameters: sTfR, CHr, LowCHr, MCVr, MCH, MCV, CH, LowCHm, and MICROm. The subjects with more advanced iron deficiency had lower values of CHr, MCVr, MCH, CH, and MCV, and higher values of sTfR, %LowCHr, %LowCHm, and %MICROm (Table 2).

Because of small numbers of subjects with iron deficiency anemia (three athletes only), the results of this condition were not compared to the other subgroups.

Decreased hemoglobin concentration and/or decreased RBC count and/or hematocrit values were observed in 6.1% of healthy subjects (without any symptoms of iron deficiency) (Table 2). These athletes were classified as subjects with symptoms of dilutional sports anemia. As compared to athletes with normal iron status they exhibited significantly (*p* < 0.001) lower values, with large effects size (d-equivalent ranging from 0.91 to 0.95) of Hb (140.7 ± 4.4 vs. 154.9 ± 7.4 g/L), RBC (4.62 ± 0.16 vs. 5.18 ± 0.25 × 10^12^/L%), and HCT (40.8 ± 1.29 vs. 45.4 ± 2.0%). Moreover, significantly (but with small effect size) higher MCH (*p* < 0.01; d-equivalent = 0.24) and lower absolute reticulocyte number (*p* < 0.001; d-equivalent = 0.29) were observed in athletes with sports anemia symptoms.

**Figure 1:**
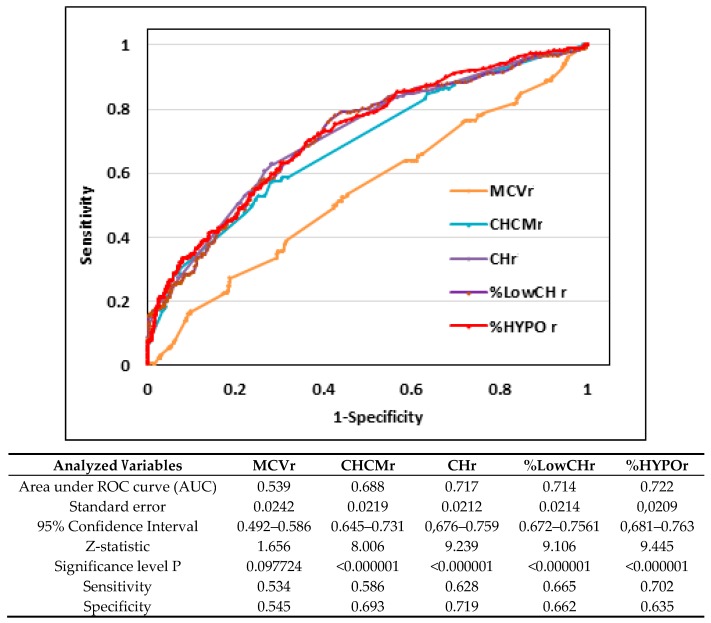
Receiver operating characteristic curve of reticulocyte indices to discriminate athletes with iron depletion (*n* = 191) from healthy athletes (*n* = 716). MCVr—mean corpuscular volume of reticulocytes, CHCMr—mean cellular hemoglobin concentration in reticulocytes, CHr—mean cellular hemoglobin in reticulocytes, %LowCHr—percentage of reticulocytes with decreased mean cellular hemoglobin content in reticulocytes %HYPOr—percentage of reticulocytes with decrease cellular hemoglobin concentration.

**Figure 2:**
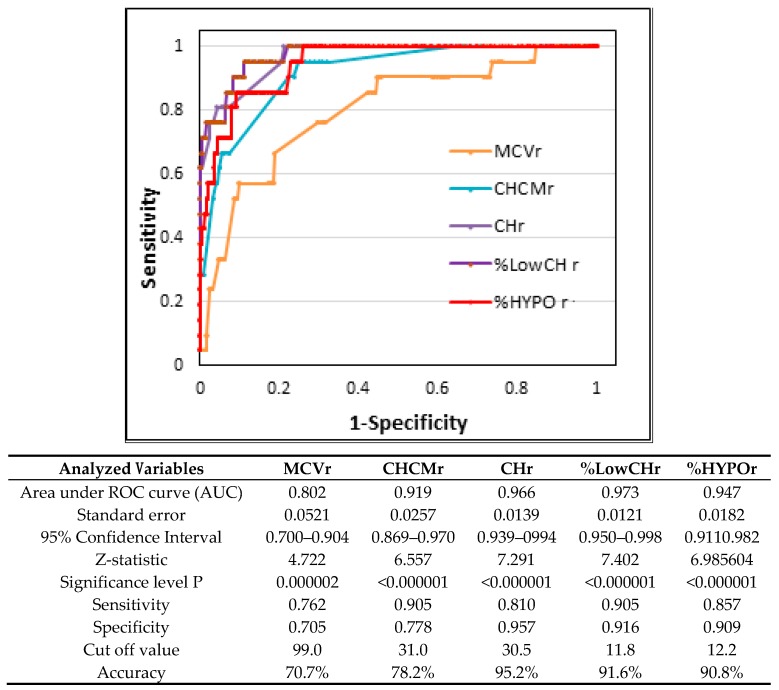
Receiver operating characteristic curve of reticulocyte indices to discriminate athletes with iron-deficient erythropoiesis (*n* = 21) from healthy athletes (*n* = 716). MCVr—mean corpuscular volume of reticulocytes, CHCMr—mean cellular hemoglobin concentration in reticulocytes, CHr—mean cellular hemoglobin in reticulocytes, %LowCHr—percentage of reticulocytes with decreased mean cellular hemoglobin content in reticulocytes %HYPOr—percentage of reticulocytes with decrease cellular hemoglobin concentration.

**Figure 3:**
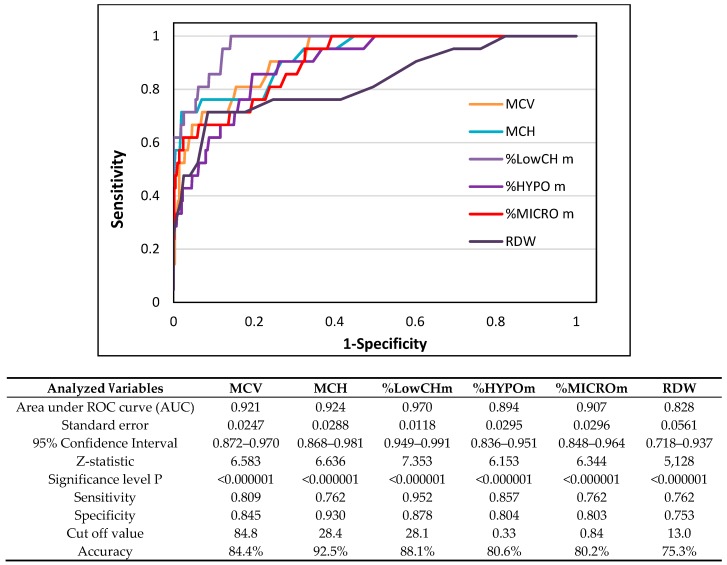
Receiver operating characteristic curve of erythrocyte indices to discriminate athletes with second stage iron deficiency (*n* = 21) from healthy athletes (*n* = 716). MCV—mean corpuscular volume, MCH—mean corpuscular hemoglobin, %LowCHm—percentage of red blood cells with decreased mean cellular hemoglobin content in erythrocytes, %HYPOm—percentage of red blood cells with decreased cellular hemoglobin concentration, %MICROm—percentage of microcytic erythrocytes, RDW—red cell distribution width.

Figure 1, Figure 2 and Figure 3 and Table 3 show the ROC-AUC analyses, which were done according to the biochemical criteria of ID to assess the efficacy of some reticulocyte and erythrocyte indices in detecting first and second stages of iron deficiency.

In the iron depletion group (Figure 1) the areas under the curve for reticulocyte parameters %HYPOr, CHr, %LowCHr, and CHCMr were comparable and were 0.722 (95% CI 0.681–0.764), 0.717 (95% CI 0.676–0.759), 0.714 (95% CI 0.672–0.756), 0.688 (95% CI 0.645–0.731), respectively. The lowest AUC, and consequently significantly lower in relation to the mentioned indices, were observed for MCVr—0.539 (95% CI 0.492–0.586). For all aforementioned parameters, both specificity and sensitivity measures were relatively low (from 0.534 to 0.719), and therefore optimal cut-off values were not calculated for this group.

In the case of stage II iron deficiency, AUC for reticulocyte parameters (Figure 2) were notably higher and were: %LowCHr—0.973 (95% CI 0.950–0.998), CHr—0.966 (95% CI 0.939–0.994), %HYPOr—0.947 (95% CI 0.911–0.982), CHCMr—0.919 (95% CI 0.869–0.970), and MCVr—0.802 (95% CI 0.700–0.904). Optimal cut-off values for detection of stage II ID were 11.8%, 30.5 pg, 12.2%, 31.0 g/dL, and 99 fL, respectively, and were calculated with the sensitivity and specificity not lower than 0.762 and 0.705 for the MCVr and as high as 0.905 and 0.916, respectively, for %LowCHr. The accuracy of the proposed cut-off values ranged from 70.7% (MCVr) to 95.2% (CHr).

The AUCs specified for mature red blood cell indices in athletes with stage II iron deficiency are presented in Figure 3 and were as follows: %LowCHm—0.970 (95% CI 0.949–0.991), MCH—0.924 (95% CI 0.868–0.981), MCV—0.921 (95% CI 0.872–0.970), %MICROm—0.907 (95% CI 0.848–0.964), %HYPOm—0.894 (95% CI 0.836–0.951), and %RDW—0.828 (95% CI 0.718–0.937). Based on high specificity and sensitivity values optimal cut-off points of these parameters for detection of iron-deficient erythropoiesis were: 28%, 28.4 pg, 84.8 fL, 4%, 0.33%, and 13%, respectively. As previously, the highest specificity and sensitivity were noted for the cut-off value of %LowCHm (0.878 and 0.952, respectively), and the estimated accuracy was 88.1%.

The values of AUC for basic erythrocyte parameters, i.e., Hb, RBC, Hct, and MCHC, in subgroups with stage I and II ID, are presented in Table 3. In subjects with iron depletion, the areas under the curve for these indices were low and ranged from 0.517 for Hct to 0.582 for MCHC. In subgroups with stage II ID, the AUC values for these basic parameters were also relatively low and were: 0.720 (95% CI 0.602–0.837) for MCHC, 0.741 (95% CI 0.645–0.838) for RBC, and 0.718 (95% CI 0.616–0.820) for hemoglobin concentration. The lowest value of AUC in this group was observed for Hct—0.615 (95% CI 0.502–0.729). Due to low specificity and sensitivity, and consequently low AUC, the cut-off values for mentioned erythrocyte indices were not calculated, even for the group with stage II ID.

## 4. Discussion

In athletes, due to the high incidence of iron deficiency, as well as its importance for general health, an accurate assessment of iron and hematological status seems to be crucial [1,40]. Seeing that these assessments may be problematic, there is a need to look for new indicators allowing for the detection of iron deficiency and reliable evaluation of hematological status, especially in physically active subjects [1,41,42,43].

Nowadays, the modern hematological analyzers give an opportunity to determine new indicators not only in reticulocytes but also in mature blood cells. These novel markers allow for a more accurate evaluation of the process of erythropoiesis in terms of the amount of hemoglobin in young and mature red blood cells, their volume, and the rate of RBC formation. So far, these indicators (especially concerning reticulocytes) have been tested mainly in patients for differentiation of IDA from anemia of chronic disease [16,21,22,44,45,46]. Their importance in detecting subclinical iron deficiencies is less known in both non-active healthy subjects [14,15,19] and athletes [25,26]. In physically active people, despite the lack of definitive evidence that subclinical ID compromises physical performance, its early detection is essential, since it helps to prevent further deterioration of iron status [1].

Although iron status is distinguished by several stages, the development of ID is a gradual process, which begins when iron stores are diminished or exhausted. Because young red blood cells mature for 1–3 days within the bone marrow and circulate for 1–2 days before becoming erythrocytes, the first adverse changes due to iron shortage should be visible in reticulocyte indices [2,44]. The results of the present study confirm this supposition since in the athletes with stage I ID, significantly lower values of CHr and CHCMr, as well as a significantly higher percentage of hypochromic reticulocytes (LowCHr and HYPOr), were observed. Moreover, our research indicated that already depleted iron stores caused changes not only in reticulocytes but also in some indices of mature red blood cells (a significantly lower value of CH (*p* < 0.001) and higher percentages of RDW (*p* < 0.001) and LowCHm (*p* < 0.001)). Despite significant differences in the above-mentioned indices, the AUC-ROC values for all reticulocyte indices (CHr, %HYPOr, %LowCHr, MCVr) were low (range: 0.539 for MCVr to 0.722 for HYPOr), so their diagnostic efficacy is not high enough. In addition, the relatively low sensitivity and specificity of all of the mentioned indices (from 0.492–0.759) did not allow us to calculate the optimal cut-off values. Similarly, the low values of AUC-ROC for basic and commonly used erythrocyte parameters (Hb, RBC, Hct, and MCHC) indicate that the diagnostic efficacy of mature red blood indices in detecting stage I iron deficiency is also not satisfactory. Thus, the present results indicate that, although in athletes with depleted iron stores visible adverse changes concern both reticulocyte and some erythrocyte parameters, the progression of these changes is insufficient for using even reticulocyte indices in the detection of this stage of ID.

In stage II ID, the prolonged absence of body iron causes a gradual and continuous increase in the number of hypochromic reticulocytes, which leads to slow, but progressive deterioration of mature red blood cell indices [2,19]. Our results confirm this process. Additionally, very high values of area under the ROC curve (>0.900) for %LowCHr, CHr %HYPOr, and CHCMr, with simultaneous maintenance of high sensitivity and specificity, are evidence of the excellent ability of these reticulocyte parameters to diagnose iron-deficient erythropoiesis in male athletes. Based on these results, it can be assumed that the athletes with CHr < 30.5 pg, LowCHr < 11.8%, HYPOr < 12.2%, and CHCMr <31 pg may be classified as subjects at high risk of at least second stage iron deficiency. This, in turn, may indicate that such athletes should be additionally diagnosed using iron-related biochemical parameters such as ferritin, TIBC, and/or sTfR.

Among the reticulocyte indices so far, the cut-off values have been determined mainly for CHr, and the obtained results are varied. There are several reasons for these discrepancies. Firstly, the CHr cut-off values were defined mostly for iron deficiency anemia (with and without inflammatory status) [11,13,16,22,44,46], and only a few studies aimed to determine them separately for subclinical iron deficiency [18,19,23]. Secondly, the determination of the threshold value for CHr was based on different criteria, i.e., the value at the 2.5th percentile [12,47], or simply, the average value [13,45,48]. Another reason for these discrepancies is the use of various hematology instruments for the measurement of cellular hemoglobin content in reticulocytes. A review of the scientific literature indicates that 28 pg is the most frequent threshold value for CHr used for IDA diagnosis in adults, although the optimal cut-off value for this condition ranges from 27 to 29 pg [11,12,20,44,46,47,49]. In our athletes with IDA (*n* = 3), the CHr values ranged from 24 to 27.4 pg, so they were below the proposed threshold value. In the IDE state, in turn, the optimal CHr cut-off value ranged from 28.0 to 30.9 pg [2,19,49,50,51], which is in agreement with our results (30.5 pg, specificity 0.957, sensitivity 0.810). The most similar optimal CHr cut-off value for stage II iron deficiency was obtained in the Toki et al. study (30.9 pg; sensitivity 92%, specificity 81%) [49] wherein the threshold value calculated by these authors at the highest specificity with simultaneous, relatively low sensitivity (68%) was lower and amounted to 28.5 pg. In our study, the same calculation of the CHr cut-off value (at the highest sensitivity, 0.997) gave the slightly higher result of 29.3 pg (data not shown). It is worth noting, however, that it came at the cost of visibly lower specificity (0.619), which in turn may increase the risk of false-positive cases in terms of detection of IDE. Thus, these results confirm that an optimal cut-off value should be set at both relatively high sensitivity and specificity for the diagnosis of iron deficiency [49].

Regarding indices of mature red blood cells in the group with stage II ID, AUC-ROC values for MCH, CH, MCV, %LowCHm, %HYPOm, %MICROm, and %RDW were high, which makes them good diagnostic markers for detecting this stage of ID. Especially %LowCHm with a high AUC value of 0.970 (CI 0.949–0.991), as well as good sensitivity (0.952) and sufficient specificity (0.878), seems to be the most promising in detection of IDE. The optimal cut-off value for this parameter in athletes with stage II ID was determined at the level of 28.1%. Unfortunately, the lack of similar studies did not allow us to compare the obtained threshold value. It is worth mentioning that the basic and commonly used erythrocyte parameters such as Hb, Hct, RBC, and MCHC had low diagnostic efficacy (AUC-ROC values from 0.615 for Hct to 0.741 for RBC); thus, they were much less informative in this stage of ID. This is partly in agreement with the results of other studies [19,52] in which moderate accuracy in diagnosing empty iron stores was observed not only for MCV and MCH but also for MCHC. So, our results demonstrate that not only reticulocyte parameters but also some new erythrocyte indices may be useful in the detection of iron-deficient erythropoiesis in male athletes.

The lack of changes in the volume of forming red blood cells (MCVr) at stage I of ID in relation to the normal iron status group, and significantly lower mean values of both MCVr and MCV, with simultaneously higher %MICROm in subjects with iron-deficient erythropoiesis than in subjects with iron depletion, indicate that the adverse changes in volume of forming and mature red blood cells start to be visible only when iron deficiency is more advanced. Furthermore, much higher areas under the ROC curve for MCVr and MCV, as well as %RDW and %MICROm in athletes with IDE versus iron depletion subgroup, also show that the volume of red blood cells begins to be diagnostic when ID is at least at stage II. All these results indicate that the earliest hematological abnormalities are mostly related to hemoglobin synthesis, wherein the volume of red blood cells is later an indicator of iron deficiency. Our results are very similar to previous observations in adolescent female athletes [25] as well as in non-athletes [14,17,52].

It is also interesting that in iron deficient athletes, adverse changes in monitored markers did not include the number of reticulocytes and RBCs, even in the second stage of iron deficiency. It suggests that with visible impairment in hemoglobin synthesis, erythropoietic activity is balanced. Higgins et al. [53] indicate that in the state of iron deficiency, the life span of RBC gets longer, and hence their breakdown is slower.

Although female athletes are particularly at high risk of iron deficiency, this problem also applies to physically active males [1]. In the present study, total iron deficiency was observed in about 23% of the subjects (*n* = 214), most of whom had stage I ID (20.5%; *n* = 191). More advanced iron deficiency, i.e., iron-deficient erythropoiesis, was found in a relatively low percentage of athletes (2.3%; *n* = 21) and only three (0.3%) subjects in the group of over 900 athletes showed iron deficiency anemia. The prevalence of iron deficiency in male athletes in previous studies ranged from 2.9 to 31.0%, [28,29,54,55,56,57,58,59,60], although the comparison of these results is difficult due to different criteria used (ferritin, or sTfR/logFerr index) as well as application of various cut-off values of ferritin (from 20 to 35 μg/L). Comparing our results to others who used the same ferritin threshold (<30 μg/L), a higher frequency of ID (27%) was observed in elite rowers and professional soccer players [60], while in large cohorts of Australian male athletes only 3–4% of subjects had a decreased ferritin concentration [58,59].

It is widely known that more advanced iron deficiency has negative effects on blood morphology indices. In athletes, however, basic morphological indices, such as Hb, RBC, and Hct, may be additionally affected by exercise-induced two-way changes in plasma volume (PV). A large increase of PV causes a decline of volume dependent hematological indices, leading to pseudo-anemia, so-called dilutional sports anemia [61]. This is a non-iron deficient condition with no negative effect on physical performance [1,62,63]. In the present study, the percentage of subjects with symptoms of sports anemia was relatively high at 6.1%. It is worth noting that Hb, RBC, and Hct were the only indicators that significantly, and with a large effect size, differentiated this group from the subjects with normal iron status (all indices dependent on plasma volume). The lack of differences in other indicators, including hypochromia indices, and an even higher mean value of MCH in the sports anemia subgroup, indicated that the analyzed new RBC indices may be helpful in distinguishing IDA and sports anemia cases, and confirm that diagnosis of sports anemia conditions in the present study was correct. Unfortunately, the lack of similar studies in athletes does not allow us to compare the frequency of this exercise-induced condition. In this context, there are mainly data concerning the impact of different types and duration of exercise on fluid homeostasis [64,65]. In one study [55], the authors just supposed that in male collegiate athletes, dilutional sports anemia could have occurred. This suggestion was based on the visibly higher percentage of low Hb concentration (9.3%) in a large sample of male athletes (*n* = 2287) than the frequency of decreased ferritin concentration in some of them (4.8% in 458 athletes). The relatively high incidence of dilutional anemia cases in our study confirmed earlier observations that indicated hemoglobin concentration might be largely dependent on the changes in PV not only in athletes [66] but also in subjects with various diseases [67], and therefore the diagnosis of anemia based on hemoglobin concentration sometimes may be erroneous.

The present retrospective study design has some strengths but also some limitations. The strengths are, the availability of data from a very large group of healthy (without symptoms of acute phase reaction) professional male athletes with a relatively high proportion of subjects with iron deficiency diagnosed based on multiple biochemical and hematological indices, allowing for accurate assessment of all three stages of ID. The performed analysis allows, for the first time, conclusions to be drawn concerning the diagnostic utility of new reticulocyte and mature red blood cell indices for two subclinical stages of ID as well as proper assessment of hematological status in athletes. In addition to several years of experience in the evaluation of iron status, all measurements were made in an accredited laboratory, which also increases the reliability of the results. Limitations include the lack of international, unequivocal threshold values for commonly used biochemical indices used for assessing iron status. Although the study concerned only healthy athletes, the impact of physical exercise on the concentration of ferritin cannot be ruled out [41]. The conclusions would certainly be more explicit if the current results could be compared with the gold standard of iron stores, which is stainable iron in bone marrow. However, due to the high invasiveness of this method, it is used very rarely. Another limitation is the lack of information concerning iron supplementation in the period preceding the study, which could impact particularly the values of reticulocyte hypochromia indices [20,51,68].

## 5. Conclusions

The present results indicate that in male athletes, the disturbances in erythropoiesis, due to iron deficiency, begin at the first stage of ID, when only ferritin concentration is decreased. These early negative changes concern, to a greater extent, reticulocytes and are related mainly to qualitative disturbances in hemoglobin production, while changes concerning the volume and amount of reticulocytes occur later. Because the changes in reticulocyte hypochromia markers are minor, their diagnostic efficacy seems to be unsatisfactory for detecting the state of iron depletion.

In more advanced iron deficiency, i.e., in IDE, indices concerning hemoglobinization in both reticulocytes and erythrocytes are characterized by a sufficiently high diagnostic utility. %LowCH in reticulocytes and erythrocytes and CHr are distinctive in this regard. At this stage of ID, reticulocyte and erythrocyte volumes start to be additionally diagnostic. These results indicate that in athletes, the markers of both hypochromia and microcythemia may be useful for the diagnosis of iron-deficient erythropoiesis.

Our results confirm that monitoring of new hematological indices related to both maturing and mature red blood cells may be useful in quickly identifying the changes in complete blood count, indicating iron deficiency, as well as in assessing its severity. The high diagnostic utility of reticulocyte and erythrocyte indices allows for screening of athletes at a lower cost, at least those with stage II ID, before biochemical tests are prescribed for further ID diagnosis. Moreover, the combined analysis of these new indicators together with routine morphological measurements and standard iron-related parameters may contribute to more accurate diagnosis of iron status, and to the early correction of ID, which may prevent further progression of ID into iron-deficiency anemia. The issue seems crucial because, despite the relatively low prevalence of anemia, the frequency of latent iron deficiency (stages I and II) concerns almost one in four of the studied athletes.

Furthermore, these new hematological parameters (especially those independent from blood volume) are helpful in distinguishing cases of true iron deficiency anemia from sports anemia conditions, which occur relatively often in this group. Therefore, they also contribute to a more accurate evaluation of hematology status in athletes.

## Figures and Tables

**Table 1:** Basic characteristics of the whole group of male athletes (*n* = 931).

Variable		Mean Value, SD and (Range)	
Age	(years)	18.7 ± 3.5	(13.0–35.1)
Body mass	(kg)	75.4 ± 11.5	(36.2–129.0)
Height	(cm)	182.5 ± 8.2	(149.5–209.7)
Athletic experience	(years)	7.7 ± 3.5	(1.0–22.0)

**Table 2:** Hematological and iron metabolism indices (mean ± SD) in healthy subjects without iron deficiency (normal iron status and sports anemia subgroups) and with iron deficiency (I and II stages of ID).

Variable	Healthy Subjects		Iron Deficiency ^b^	
	Normal Iron Status	Sport Anemia ^a^	Iron Depletion	IDE
	(*n* = 659)	(*n* = 57)	(*n* = 191)	(*n* = 21)
Ferritin (µg/L)	64.1 ± 32.0	61.0 ± 22.8	22.6 ± 4.9***	13.5 ± 7.3***
sTfR (mg/L)	4.9 ± 1.2	4.98 ± 1.13	5.56 ± 1.29***	9.35 ± 2.84***^^^^^
TIBC (µg/dL)	319.6 ± 32.9	314.4 ± 27.7	347.4 ± 35.8**	394.6 ± 41.9***^^^^
Iron (µg/dL)	102.3 ± 39.9	89.4 ± 35.5*	89.7 ± 37.5***	63.6 ± 32.0***^
RBC (×10^12^/L)	5.18 ± 0.25	4.62 ± 0.16***	5.20 ± 0.30	5.39 ± 0.27**^
Hct (%)	45.4 ± 2.0	40.8 ± 1.29***	45.0 ± 2.3*	44.3 ± 1.9*
Hb (g/L)	154.9 ± 7.4	140.7 ± 4.4***	152.0 ± 8.4***	147.5 ± 6.9***
MCH (pg)	29.9 ± 1.11	30.5 ± 1.29**	29.3 ± 1.24***	27.4 ± 1.41***^^^^^
MCHC (g/L)	341.3 ± 10.5	345.3 ± 13.5*	338.4 ± 10.8***	333.1 ± 10.6***
MCV (fL)	87.8 ± 2.8	88.4 ± 3.8	86.6 ± 3.4***	82.2 ± 2.9***^^^^^
CH (pg)	29.9 ± 0.99	30.3 ± 1.09*	29.2 ± 1.11***	27.3 ± 1.55***^^^^^
RDW (%)	12.7 ± 0.43	12.7 ± 0.45	13.0 ± 0.49***	13.5 ± 0.82***
HYPOm (%)	0.24 ± 0.34	0.23 ± 0.26	0.55 ± 0.73***	1.72 ± 2.28***^^^^
Low CHm (%)	18.5 ± 7.3	16.8 ± 7.1	25.8 ± 10.1***	44.7 ± 14.5***^^^^^
MICROm (%)	0.61 ± 0.33	0.57 ± 0.33	0.83 ± 0.47***	1.99 ± 1.41**^^^^^
Reticulocytes (%)	1.46 ± 0.34	1.44 ± 0.37	1.42 ± 0.35	1.41 ± 0.26
Reticulocytes (10^9^/L)	75.5 ± 18.4	66.6 ± 17.3***	73.6 ± 18.3	76.1 ± 15.7
MCVr (fl)	100.8 ± 2.7	101.7 ± 3.2	100.6 ± 2.8	97.7 ± 2.76***^^^^^
CHCMr (g/dL)	32.1 ± 0.99	32.1 ± 0.90	31.3 ± 1.11***	30.0 ± 1.18***^^^^^
CHr (pg)	32.3 ± 1.07	32.4 ± 1.09	31.4 ± 1.22***	29.2 ±1.48***^^^^^
Low CHr (%)	5.9 ± 3.7	5.7 ± 3.9	9.8 ± 6.1***	26.4 ± 16.3***^^^^^
HYPOr (%)	5.7 ± 4.7	5.6 ± 3.7	11.1 ± 8.7***	25.8 ± 17.4***^^^^^
Body Iron (mg/kg)	7.55 ± 1.86	7.40 ± 1.63	3.57 ± 1.44***	−0.67 ± 3.11***

^a^ Difference vs. normal iron status analyzed by the U Mann-Whitney test, ^b^ Difference vs. normal iron status tested by the Kruskal-Wallis test followed by Dunn’s post hoc comparisons; significantly different from the respective value in the group with normal iron status. *p < 0.05; **p < 0.01; ***p < 0.001; significantly different from the respective value in the group with I stage of iron deficiency. *^p* < 0.05; ^*^p* < 0.01; *^^^p* < 0.001; IDE—iron-deficient erythropoiesis, Hb—hemoglobin concentration, Hct—hematocrit, RBC—red blood cell count, MCHC—mean corpuscular hemoglobin concentration, MCV mean corpuscular volume, MCH—mean corpuscular hemoglobin, CH—mean cellular hemoglobin content in erythrocytes, %HYPOm—percentage of red blood cells with decreased cellular hemoglobin concentration, %LowCHm—percentage of red blood cells with decreased mean cellular hemoglobin content in erythrocytes, %MICROm—percentage of microcytic erythrocytes, RDW—red cell distribution width, CHr—mean cellular hemoglobin in reticulocytes, #RET—absolute number of reticulocytes, %RET—absolute reticulocyte count as a percentage, CHCMr—cellular hemoglobin concentration mean in reticulocytes, MCVr—mean corpuscular volume of reticulocytes, %HYPOr—percentage of reticulocytes with decrease cellular hemoglobin concentration, %LowCHr—percentage of reticulocytes with decreased mean cellular hemoglobin content in reticulocytes, sTfR—soluble transferrin receptor concentration, TIBC—total iron binding capacity value.

**Table 3:** Performance of basic erythrocyte parameters: Hb, RBC, Hct, and MCHC in the diagnosis of I and II stage of iron deficiency in male athletes.

	Iron depletion			IDE		
Analyzed Variables	AUC of ROC	(95% CI)	*p*-value	AUC of ROC	(95% CI)	*p*-value
Hb	0.564	0.518–0.610	0.006366	0.718	0.616–0.820	0.00065
RBC	0.558	0.512–0.605	0.012606	0.741	0.644–0.838	0.000161
Hct	0.517	0.470–0.563	0.470684	0.615	0.502–0.729	0.070867
MCHC	0.582	0.537–0.628	0.0005	0.719	0.602–0.837	<0.0006

Hb—hemoglobin concentration, Hct—hematocrit, RBC—red blood cell count, MCHC—mean corpuscular hemoglobin concentration.
